# A qualitative study about the mental health and wellbeing of older adults in the UK during the COVID-19 pandemic

**DOI:** 10.1186/s12877-021-02367-8

**Published:** 2021-07-26

**Authors:** A. R. McKinlay, D. Fancourt, A. Burton

**Affiliations:** Research Department of Behavioural Science and Health, Institute of Epidemiology & Health Care, University College London, 1-19 Torrington Place, London, WC1E 7HB UK

**Keywords:** Older adults, Mental health, Social support, Covid-19

## Abstract

**Objectives:**

The objective of this study was to examine factors that threatened and protected the wellbeing of older adults living in the UK during social distancing restrictions due to the COVID-19 pandemic.

**Methods:**

Semi-structured telephone or video interviews with 20 adults aged over 70. Purposive sampling methods were used to increase diversity within the group. Transcripts were analysed using reflexive thematic analysis.

**Results:**

Participants described potential threats to their wellbeing during the pandemic, including fears for mortality, grieving normal life, and concerns for the future. Participants also described activities and behaviours that helped to protect their mental health, including adopting a slower pace of life, maintaining routine, socialising, and using past coping skills. Many participants drew on their resilience and life experience to self-manage fear and uncertainty associated with the pandemic, using their time during lockdown to reflect or organise end-of-life affairs.

**Discussion:**

This study provides UK-based evidence that while some older adults experienced challenges during the first wave of COVID-19, many were resilient throughout social distancing restrictions despite early reported concerns of mental health consequences among the older adult population. Our findings highlight the importance of maintaining access to essentials to promote feelings of normality and use of social support to help reduce uncertainty in times of pandemics.

**Supplementary Information:**

The online version contains supplementary material available at 10.1186/s12877-021-02367-8.

## Introduction

Existing concerns about the wellbeing of older adults were exacerbated when severe acute respiratory syndrome coronavirus 2 (SARS-CoV-2) was declared a pandemic by the World Health Organisation on March 11th, 2020 [39]. Older adults were identified as especially vulnerable to the virus with high rates of fatalities [17], particularly in some residential care homes [9, 35] during the first wave of the virus [23]. Hospitalisation rates were high among those living with long term conditions (LTCs) [10, 17], many of which affect the older adult population [12, 30]. The UK government imposed their first social distancing restrictions on March 23rd, 2020, where adults over the age of 70 were required to self-isolate and “lockdown” at home for 3 months to reduce their infection risk.

Drawing on evidence of negative psychological responses observed during previous epidemics [5], concerns rose among stakeholders at the start of the pandemic that there would be adverse effects of the COVID-19 pandemic on mental health and wellbeing. Whilst under usual circumstances, older adults do tend to experience psychosocial wellbeing that is equal or better than that of younger age groups [11], it was predicted that due to the specific isolation rules for older adults and their heightened risk from the virus, psychosocial consequences such as loneliness would be exacerbated in older age groups [21], leading to negative effects on mental and physical health [22, 34]. At a population level, mental health during the COVID-19 pandemic was negatively impacted [42], but evidence suggests older adults on average experienced more stable and less negative outcomes compared with other subgroups [20, 38]. It is presently unclear why this was, or what underlying factors accounted for the experiences reported by older adults during lockdown.

Several theories could help to explain the apparent psychological resilience of older adults during the pandemic. Offers of support from social contacts [25], a stable living environment [6, 7], cohabiting with others [19], and financial security [20] may have helped protect many in this group against adverse effects of social distancing measures by providing a psychological buffer against distress. Additionally, older adults may draw on previous life experiences to perceive a greater sense of coherence in the events of the pandemic. Sense of coherence theory incorporates comprehensibility (ability to understand and integrate), manageability (ability to navigate and manage) and meaningfulness (sense making) in relation to interpretation of a new health threat [1]. It has been shown to support better navigation of life stressors [1] and is a strong predictor of health status among older adults [16]. Life wisdom accumulated by older age has also been found to increase the use of problem-focused coping skills, which may protect against distress [14]. However, whether factors such as these do indeed explain the responses amongst older adults remains unexplored.

Understanding the factors that are transferable across age groups is essential in developing future interventions and policy for those most at risk of harm due to social distancing measures during the pandemic. Further, whilst the average mental health symptom scores and wellbeing levels of older adults have been better than amongst younger age groups during the first wave of the pandemic in the UK [15], this does not necessarily imply that older adults were psychologically unaffected. Therefore, this study explored in detail the experiences of older adults living in the UK, with two specific research questions: (1) How was the mental health of older adults affected during the pandemic? (2) What factors have protected mental health in older adults during this time?

## Methods

### Study design

This research was undertaken as part of the COVID-19 Social Study (CSS) that began on March 21st 2020 [6, 7], which is the largest UK panel survey study on social life during the COVID-19 pandemic. The overall aims of this work are to explore the psychosocial impact of the pandemic among people living in the UK. In this qualitative substudy, conducted separately from the CSS survey, we elicited perspectives of older adults through qualitative interviews, which were carried out from May until September in 2020. We deployed phenomenological methodology to interrogate the data and focus on individual accounts of experience, coupled with reflexive thematic analysis techniques for analysing and framing the research data. The University College London Ethics Committee reviewed and approved this study (Project ID: 14895/005). Content in the following sections are informed by the COREQ reporting guidelines [37].

### Recruitment

Eligibility criteria included: aged 70 years or older, and the ability to speak English sufficiently to understand the study participant information sheet and consent form. We recruited participants by listing the substudy in the CSS newsletter (reaching 3919 subscribers), social media, and through two community organisations who circulated study information within their networks. We did not record response rates during recruitment. People interested in participating were asked to contact the research team directly via email. In order to understand a range of individual experiences, we screened for characteristics (such as gender, ethnicity, educational level) based on previous findings highlighting how some demographics factors have been associated with adverse mental health during the pandemic [15]. Thus, we used purposive sampling methods to ensure that 20 adults aged over 70 were selected from diverse backgrounds in terms of gender, ethnicity, marital status, and living situation. Recruitment ended after 20 one-off interviews, as the lead author AM identified no new themes during the analysis.

### Procedure

A researcher (AM or AB) responded to expressions of interest in the study with further details﻿ about the study and an invitation to ask additional questions. All participants then provided written informed consent prior to attending a remote interview by telephone or video call. Participants were offered a £10 shopping voucher as an expression of gratitude. A team of female, postgraduate-level, qualitative healthcare researchers conducted all interviews (AM, AB, LB, AR, SC). No researcher had prior relations with any research participant. Interview times ranged from 16 to 85 min and lasted for 50 min on average. A complete interview guide can be found in Supplementary File 1. In brief, interview topics included: normal life before the pandemic, understanding of social distancing guidelines, social life, mental health, and prospection (for question examples, refer Table 1). Interview guide questions and prompts were developed based on concepts from social integration and health theory [2] and Antonovsky’s Sense of Coherence theory [13]. For example, *“Has the pandemic meant that you have any worries for the future? How are these different from the worries you had before?”* Although all general topics were discussed during interviews, not all questions or prompts were used or indeed relevant to each participant’s unique circumstances. Interviewers were guided about the questions and prompts to use according to participant responses.

**Table 1:** Example of interview guide questions

Questions	
1. How would you describe your social life now that social distancing measures have been brought in because of Covid-19?	
2. Have you been able to stick to the social distancing advice that has been given to your group?	
3. How do you feel about the changes that have been brought about by Covid-19?	
4. Have they had any impact on your mental health or wellbeing?	
5. Has the pandemic meant that you have any worries for the future?	

### Data analysis

Researchers audio recorded the interviews with consent from participants, which were then transcribed verbatim by a professional transcription service. All transcripts were manually checked for anonymity after transcription before importing into Nvivo version 12 for analysis. Transcripts were not returned to participants for comment or correction, nor did they provide feedback on the findings. For consistency of coding approach, AM and AB double coded 3 transcripts at the start of data analysis and discussed issues of salience raised by participants. We did not calculate the intercoder reliability or quantify agreement during this stage [29], but rather focused on the impressions that both researchers had on topics of importance when coding the same passage of text. The lead researcher (AM) used an inductive and deductive, reflexive thematic analysis approach, informed by Braun and Clarke [3, 4]. An initial coding framework was established from the topic guide, which was formulated based on supporting theory regarding social network structure, social ties, social support (i.e., [1, 2]). This framework was applied to each transcript through line-by-line coding, then the framework was updated with new codes as AM identified new concepts in the transcripts described by participants. Themes and subthemes were therefore developed based on participant narratives, and these were presented to the CSS research team on 3 occasions throughout the analysis stages for formative feedback.

## Results

Of those who agreed to take part, 9 participants were women and 11 men, with an average age of 79 (Table 2). Fourteen participants reported having a physical health condition, including hypertension, diabetes, arthritis, high blood pressure and cancer. Two participants had an anxiety-related mental health condition diagnosed prior to the pandemic, and 3 said they had caregiving responsibilities for a spouse or family member.

**Table 2:** Participant characteristics

Demographic factors	Sample *n* = 20
**Age bands**	
70s	10
80s	9
90s	1
**Ethnicity**	
White British	16
Asian Other	2
Other (Myanmar)	1
White Other	1
**Relationship status**	
Married/Partnership/Cohabiting	10
Widowed	6
Separated/ Divorced	3
Single (never married)	1
**Employment**	
Retired	16
Working part time	2
Self employed	2
**Living situation**	
With partner/spouse	9
Alone	8
With family	2
With friends	1

Participants reported varied and nuanced experiences from the outset of the COVID-19 pandemic. We therefore generated two overarching themes, each with 4 subthemes. Many participants described potential threats to their wellbeing, including fears for mortality, grieving normal life, restricted access to support, and concerns for the future. However, many spoke of how they protected mental health in response to pandemic-related uncertainty, including adopting a slower pace of life, maintaining routine, socialising, and using past coping skills.

### Potential threats to wellbeing

Some participants described a transient period of uncertainty at the start of the first UK lockdown, associated with nervousness and lack of sleep that resolved quickly. Many were understandably concerned about the impact the pandemic was having on their end-of-life experience and consequences for the rest of the world (Table 3). Overall, the government restrictions were described as “fair enough” and “required” by many; however, a commonly reported concern among participants was a fear of needing healthcare assistance during the COVID-19 lockdown. Some participants were worried about catching COVID-19 and additional risks due to their age, ethnicity, and medical history. Others said that lockdown measures meant they were unable to engage in activities that usually formed part of their self-care routine.

**Table 3:** Commonly reported themes about threats and benefits to wellbeing during lockdown

Theme	Subtheme
**Potential Threats to Wellbeing**	• Concerns about end-of-life, ageing, and mortality • Thinking about end-of-life concerns, worries about ageing and frailty
	• Grieving the loss of normality • Feeling life is on hold, craving normality, finding the state of the world upsetting
	• Healthcare concerns • Fear of hospitalisation, fear of seeking help due to perceived lack of service availability, fear of leaving the house due to COVID
	• Unable to engage with activities that protect wellbeing • Loss of leisure, lack of routine
**Protective Activities and Behaviours**	• Slowing the pace of life • More time for exercise and new hobbies, time for introspection, and organising affairs
	• Benefits of routine and social responsibility • Feeling “needed” and helping others, keeping busy with social obligations
	• Social interaction and support • Connecting with others, reciprocal offers of support
	• Utilising skills, experience and resources to cope • Using past coping skills and experience, accustomed to isolation, accessing practical resources

#### Concerns about end-of-life, ageing, and mortality

More than half of the group spoke about how the pandemic caused them to think about their experience of end-of-life, ageing, and mortality.

*‘It’s just this idea of all of a sudden realising that I’m getting really old. I think that may be the biggest thing, and it’s a combination of getting really old, and the pandemic is probably accentuating it a bit.’ p10, female, aged 75-79*

Those who were retired were particularly worried about the physical or cognitive impact of lockdown on their experience of ageing.

*‘I thought I’m going to be forced into being isolated at home. Can’t go to the gym, can’t go out walking, I’m going to physically deteriorate. And I really was quite scared about that.’ p1, male, aged 80-84*

Some planned for the possibility of becoming unwell from COVID. For instance, one participant had instructed his family to “stay away” should he become gravely ill, to protect them from the virus. For several participants, a reminder of their own mortality risk came from knowing someone who had passed away from COVID:

*‘We’ve had one friend who was in his sixties… Suddenly went into hospital went on a respirator and sadly he died. He’s the only person we know who has directly been affected by it. It hits you and it makes you realise your own mortality. Especially when they keep saying it affects older people worse, so you do worry.’ p13, male, aged 75-79*

Several participants described concerns about their perceived vulnerability to COVID due to their age, ethnic group, or pre-existing health concerns:

*‘It is scary for us at our age. The thought of getting COVID, that really frightens me and frightens me for anybody close to me that if they got it. It really terrifies me. So, we have been very, very careful.’ p11, female, aged 70-74*

#### Grieving the loss of normality

Understandable emotional responses and a longing for normality were frequently described by participants. Some felt the activities they previously enjoyed, like travelling, going to the theatre, or “hitting a tennis ball,” would never return to the normal they were used to. Others said they felt their life was on hold until the virus was under control or a vaccine was introduced.

*‘The new normal is not going to be at all like the old normal, I don't think. We won’t really be able to live the kind of life that we lived before until there’s a vaccine, and it looks as though the vaccine is going to be a very long way off.’ p4, female, aged 70-74*

Some said they felt grief about the impact COVID was having on the world, particularly regarding death, hardships, and suffering of others.

*‘I knew of the wars and the disease and the hunger, but I think COVID has just put a whole blanket round the lot of it and makes it so immense, the state of the world. The horrible state of the world and that is very depressing when you think about it.’ p19, female, aged 80-84*

Several said they did not think a COVID vaccine would help life “go back to where we were before.”

*‘Whatever happens, even if a vaccine comes, we will never return to shaking strangers’ hands.’ p16, male, aged 75-79*

#### Healthcare concerns

Concerns about catching COVID were variable among the group, but many were more fearful of being hospitalised for any reason because they believed they were at increased risk of death.

*‘A lot of people are scared stiff of catching [COVID], I’m not. The only thing I’m scared of is being carted off to a hospital. I want to die peacefully at home, and I would happily do that any night.’ p8, male, aged 90-94*

Those living alone in particular worried about the lack of available health services during lockdown, should anything “go wrong” with their health independently from COVID.

*‘A friend of mine has just been diagnosed with breast cancer. She’s had to wait about nine weeks for her op… so you worry about if something like that happened to me, would I get the medical attention I need?’ p12, female, aged 75-79*

The potential health threat of COVID meant some participants were scared to leave the house.

*‘I do feel that perhaps I should be going out more and that sort of thing, but myself and many, many, almost all my friends say that they are very scared to go out.’ p2, female, 70-74*

Several participants had decided to self-isolate before the national restrictions were introduced, mainly due to concerns about age-related vulnerability and pre-existing health conditions.

*‘Come mid-March when it all happened… we decided ourselves to lockdown before other people did… I’m over 75 and I’ve got blood pressure controlled by medication but I’m over 75. My BMI was over 30… So, I was worried and we were worried. So, we totally shut down.’ p16, male, aged 75-79*

#### Unable to engage with activities that protect wellbeing

Due to social distancing and travel restrictions, some participants were unable to engage in activities such as weekly religious ceremonies, theatre groups, and sports. Although some activities were able to be undertaken online, this was not always possible.

*‘Since COVID, [community activities have] all closed down. Well yes, the book club totally because we can’t discuss books over the phone and also people are of an age where you can’t do social media, whatever you call it.’ p19, female, aged 80-84*

Several participants commented on the consequences of an abrupt change in routine on their wellbeing during lockdown.

*‘That was the first thing that hit me, boredom. I had no idea what the hell am I going to do next, because I was used to a routine and suddenly the routine was completely disrupted…Now suddenly I had nothing to do and I was really lost. I was walking round the house like a bloody zombie trying to find something to do.’ p15, male, aged 80-84*

### Protective activities and Behaviours

Despite voicing threats to wellbeing, many participants were positive in reflecting on their lockdown experience. This was attributed to a slowed pace of life, maintaining a routine, using coping skills and resources, and accessing social support (Table 3).

#### Slowing the pace of life

The most commonly reported experience during lockdown was feeling like the pace of life had slowed on an individual and societal level, with more time alone to reflect. Although some participants had described a loss of leisure during lockdown, many had found time for new hobbies, reading, crafts, gardening, and learning a new language.

*“Sometimes I wake up in the morning, and I think, oh, it’s another day in lockdown, but I think… there are some little positive benefits…Before lockdown, we were all rushing around doing lots of things, and now we’ve had to slow down. And actually, slowing down has been quite nice. And we’re living in the kind of retirement now that, maybe, our grandparents might have lived, when you just cultivate your garden and do your knitting and crochet... But just generally living a slower pace of life.“ p4, female, aged 70-74*

Others felt that being required to stay at home presented an opportunity to focus more on their health by going for regular walks and taking up new forms of physical activity. For some, this was the first time in decades they had been so physically active.

*“I’ve now started to ride a proper bike as well. I live in a Close, so we don’t get any through traffic and I can cycle around that Close and I do a few laps. But I haven’t ridden a bike for 60 years.” p1, male, aged 80–84*

Half of the group said the slowed pace of life gave more time for introspection: *“I’m not rushing around so much anymore, it’s given me the time and the opportunity to notice small things.” p4, female, aged 70–74.* In particular, many women in the group said they reflected on their life differently and in a more meaningful or positive way than before. Some used this process of reflection to think about the changes they would make to their lives as a result of their pandemic experience.

*‘Having grown quite a lot, I feel quite positive about that. I also think I’m going to try and, maybe, achieve more things when I come out of this [lockdown]. I think when you retire, and as you get older, you become very comfortable in your life. I think, perhaps, I was a bit too comfortable. I need to get out and be more proactive.’ p6, female, aged 70-74*

#### Benefits of routine and social responsibility

Nearly half of participants said that maintaining a routine and sense of purpose was important for their wellbeing during the COVID-19 lockdown: “*You have to have a purpose you see. I think mental resilience is all about having a sense of purpose.” (p15, male, aged 80–84).*

Many female participants said they experienced meaningful benefits from social responsibilities, such as cooking a meal for family, phoning friends to check in, or caring for a pet:

*‘The important thing is to have the necessity to do things. Whether it is to get in touch with people, to write a piece of something… Obligations are a good source of maintaining ones feeling of self-worth, if you like. So I think it’s very important to make sure that whatever it is, even though you may feel oh what a nuisance I’ve got to do that, the very fact of having to do it is a great psychological benefit.’ p3, female, aged 70-74*

#### Social interactions and support

The nature of socialising had changed since the start of lockdown for many but not all participants. Several said they were socialising to try to carry on *“life as normal”*, particularly keeping in regular contact with family. For some, this resulted in strengthened relationship bonds and connectedness:

*‘I think it has made me and my husband stronger really. We’ve never spent as much time together actually… I think we’ve coped with the shopping and organising that. And we’ve been baking together, we’ve never done things like that. And we took it in turns to cook and tidy up after. We have done really well together. I’m really proud of us.’ p11, female, aged 70-74*

#### Utilising skills, experience and accessing practical resources to cope

Participants who had used mental health services in the past spoke of utilising the skills they had previously learned to help cope with the COVID-19 crisis, including use of mindfulness and meditation.

*‘I had a wonderful counsellor who I saw about once a year, and she would set me on the right path. And eventually, after many years of trying, I found a mindfulness and meditation book, about the middle of last year… so I feel that that has been a great help to me. Usually I try in the morning and certainly in the evening, before I go to bed, I do some meditation.’ p2, female, 70-74*

Others described experiences of hardship in the past that they used as a comparison with COVID times, such as living through war, displacement, and illness:

*‘I was diagnosed with what they call non-invasive bladder cancer… Having gone through the concern of something like that, perhaps Covid, you know, you put it into perspective.’ P13, male, aged 75-79*

For some who lived alone, they spoke of being accustomed to isolation long before the pandemic arrived: *“I’m a fairly sort of isolated person anyway.” p1, male, aged 80–84*. Several said they were accustomed to being alone due to widowhood or retirement, and therefore lockdown did not prompt a dramatic change in their daily living or social life:

*‘I’ve been retired for a nice long time… So, in many ways the lockdown, on one side it hasn’t impacted a great deal, because I was used to being at home and certainly over the past two years to being home alone.’ p20, male, aged 80-84*

Participants frequently mentioned their access to practical resources and basic necessities that helped reduce uncertainty, such as access to online shopping for home food deliveries and offers from others to drop off medication. Such arrangements had been made during lockdown, with additional support offered by family, friends, and neighbours.

*‘I’ve had online shopping every week since lockdown and I haven’t been to any shop. Prescriptions were delivered and anything I wanted, my daughter would fetch.’ p18, female, aged 80-84*

## Discussion

In this study, we sought the views of older adults living in the UK about factors that threatened or protected their mental health and wellbeing during the COVID-19 pandemic. Our study identified understandable emotional responses to the pandemic including fears relating to the virus, the future, and mortality. These are justified in the face of unprecedented circumstances, such as those brought about by COVID-19 [28]. Overall, older adults mostly described engaging with activities and behaviours that helped to protect their mental health and could explain their improved wellbeing relative to other age-groups. For the most part, participants enjoyed feeling less social pressure and having more time for their hobbies. Similarities in experience were drawn by this group between a slower pace of life germane to retirement and day-to-day realities of the COVID-19 lockdown. As described by older adults in Japan [36], COVID-19 restrictions introduced comparatively few changes to daily life compared with other groups. Those who experienced greater daily changes and uncertainty, such as parents of young children, working age adults and those affected by financial difficulties, have reported greater levels emotional distress during the pandemic [32]. Fewer changes and transitions experienced by older adults may therefore explain some of the differences observed in levels of psychological distress.

Congruent with international research [25], many participants began to self-isolate earlier than guidance required and perhaps consequently, practical arrangements were in place for access to essentials from the outset of lockdown (for instance, food and medication deliveries offered to people aged over 70), resulting in greater sense of coherence of COVID-19 as a potential health threat. Being at home early meant less opportunities for virus exposure, perhaps reducing fear of virus transmission and creating an environment of stable predictability (comprehensibility). Many older adults were offered online shopping slots or received offers of help from friends, family or neighbours for medication collection, meaning access to supplies was not restricted (manageability). And for the most part, older adults in our study made sense of the pandemic with reference to previous major events in their lives, such as war and displacement. Explanations about their behaviours and adherence during this time was described as behaviour “for the greater good” for the rest of society (meaningfulness).

### Factors that threatened mental health and wellbeing during COVID-19

Given early evidence publicised on mortality risk for older adults [41], it is unsurprising participants frequently discussed concerns about their end-of-life. Studies have shown an association between social isolation and reduced physical performance, [31] causing concern among some participants in our study, with many taking extra steps to preserve their activity levels. While this may have provided positive health benefits in the short term, of most concern is the fear many participants described in leaving the house to access routine or preventative health care, which may have longer-term implications for public health services. Aligned with international research [24], participants in our study also worried about the impact of COVID-19 on the world and spoke of the impact this had for their wellbeing on a daily basis. Feelings of grief and loss were frequently reported and will likely be felt across many societies in response to the pandemic.

### Factors that protected mental health and wellbeing during COVID-19

Quantitative data collected during the first UK lockdown suggests that those with restricted finances and access to basic needs experienced higher levels of adversity during the first wave of the pandemic [40]. Many participants in our study reported having access to basic supplies (via online shopping slots and medication deliveries) and high levels of perceived social support, which may have helped to create a buffer against stress and uncertainty. National averages showed infrequent experiences of loneliness among older adults during the pandemic [26], which may be explained by our finding that participants engaged frequently in online methods of interaction, spent time with pets, and/or had regular remote “check-ins” with friends and family to mitigate against loneliness. As such, the heightened concern about loneliness in this age group early on in the pandemic may have led older adults and those around them to proactively take steps that helped prevent these experiences in many individuals. Indeed, many participants reported enhanced feelings of connectedness with social contacts throughout the lockdown, which can prevent isolation and protect against emotional distress [34]. However, a small number of participants did not feel connected, particularly those who had been separated from their family because of the pandemic, highlighting the difficulties experienced when such support was not available.

### Implications

This study highlights a number of important implications. First, the potential threats to wellbeing amongst older adults require further consideration as they have implications for the immediate future and for future pandemics. In trying to remove barriers to healthcare access, supporting older adults in engaging with telecare may be a helpful alternative for some health concerns. However, in our CSS work involving people with mental health conditions in the UK, we found service users felt this was an unhelpful substitute [8, 9]. Future research must address indirect health consequences of the pandemic resultant of delayed or diminished access to healthcare during the lockdown. Second, as discussed elsewhere [18], interventions to mitigate the impact of prolonged isolation on experiences of grief are warranted. Grief can prompt search for meaning and seeking out others with similar experiences. Clinicians play a role in supporting people in processing their grief associated with COVID-19, but spaces online and within groups may also facilitate healing from loss experienced during the pandemic [18]. Schemes such as social prescribing could be deployed to support older adults psychosocially, and may provide additional support in the aftermath of COVID-19 [33]. Finally, it is evident that forward-planning by families and communities to address initial concerns about older adults during the pandemic played an important role in supporting their coping and buffering against loneliness, isolation, and uncertainty. For future pandemics, such a response is again encouraged. In particular, interventions that bolster feelings of certainty and connectedness may serve as helpful targets for those experiencing pandemic-related distress.

### Strengths and limitations

A strength of this research is that data were collected from participants via purposive recruitment throughout the first UK lockdown and as restrictions began to ease before the second wave. However, findings must be interpreted cautiously. Our participants were generally healthy, with well-established social networks, living in the community, and predominantly without solo caregiving responsibilities. Therefore, their experiences are not likely to be representative of those living with serious health concerns, who may be more likely to have experienced distress during the pandemic [27]. We conducted interviews via video call or telephone, which meant being able to capture experiences safely amid restrictions, but also means that those without access to the internet or telephone would not have had equitable access to participate and may have faced additional challenges. We also did not collect data on, or sample based on socioeconomic status or previous COVID-19 infection. To our knowledge, no participant had experienced a confirmed diagnosis. Future studies are needed to ascertain how older adults who experienced COVID-19 were impacted psychologically [26].

## Conclusions

Contrary to early concerns at the start of the pandemic, the mental health of older adults fared well compared with other age groups, and this study adds to the literature on this topic by providing evidence as to why these results may have been found. Overall, many participants described their experience of lockdown as a time for reduced social pressures and increased opportunities for personal growth. However, this group experienced challenges, particularly among those who were concerned about staying well, advancing frailty, or hospitalisation risk. This research therefore highlights the importance of nuance when considering the relative better experiences of older adults. It also provides valuable insight into factors that protected wellbeing of older adults during the COVID-19 pandemic, which may be utilised by policy makers to support at-risk groups who have experienced psychological hardship during the crisis, including timely access to essential supplies, communicating offers of help to improve perceived support, and providing structure and routine in times of uncertainty.

## Supplementary Information

**Additional file 1.** Interview topic guide: Adult groups.

## Data Availability

The datasets generated and analysed during the current study are not publicly available and are not available from the corresponding author on request due to reasons concerning participant privacy and confidentiality.
